# Supplementary material for: Flexible Work: Opportunity and Challenge (FLOC) for individual, social and economic sustainability. Protocol for a prospective cohort study of non-standard employment and flexible work arrangements in Sweden
Source: BMJ Open. 2022 Jul 11;12(7):e057409. doi: 10.1136/bmjopen-2021-057409 (PMC9274509; doi:10.1136/bmjopen-2021-057409)
Supplement: Author's manuscript [file bmjopen-2021-057409.draft_revisions.pdf]

BMJ Open is committed to open peer review. As part of this commitment we make the peer review history of every article we publish publicly available.

When an article is published we post the peer reviewers' comments and the authors' responses online. We also post the versions of the paper that were used during peer review. These are the versions that the peer review comments apply to.

The versions of the paper that follow are the versions that were submitted during the peer review process. They are not the versions of record or the final published versions. They should not be cited or distributed as the published version of this manuscript.

BMJ Open is an open access journal and the full, final, typeset and author-corrected version of record of the manuscript is available on our site with no access controls, subscription charges or pay-per-view fees (http://bmjopen.bmj.com).

If you have any questions on BMJ Open's open peer review process please email info.bmjopen@bmj.com

BMJ Open

FLexible work, Opportunity and Challenge (FLOC) for individual and organizational sustainability: Protocol for a prospective cohort study of non-standard employment and flexible work arrangements in Sweden

Journal:	*BMJ Open*
Manuscript ID	bmjopen-2021-057409
Article Type:	Protocol
Date Submitted by the Author:	14-Sep-2021
Complete List of Authors:	Svensson, Sven; University of Gävle, Department of occupational health science and psychology<br>Hallman, David; University of Gävle, Department of occupational health science and psychology<br>Mathiassen, Svend ; University of Gävle, Department of occupational health science and psychology<br>Heiden, Marina; University of Gävle, Department of occupational health science and psychology<br>Fagerström, Arne; University of Gävle, Faculty of Education and Business Studies<br>Mutiganda, Jean Claude; University of Gävle; University of Gävle, Faculty of Education and Business Studies<br>Bergström, Gunnar; University of Gävle, Department of occupational health science and psychology
Keywords:	EPIDEMIOLOGY, OCCUPATIONAL & INDUSTRIAL MEDICINE, SOCIAL MEDICINE

SCHOLARONE™
Manuscripts

FLexible work, Opportunity and Challenge (FLOC) for individual and organizational sustainability: Protocol for a prospective cohort study of non-standard employment and flexible work arrangements in Sweden

Sven Svensson*[1], David Hallman[1], SvendErik Mathiassen[1], Marina Heiden[1], Arne Fagerström[2], Jean Claude Mutiganda[2], Gunnar Bergström[1]

* Corresponding author:

Sven Svensson
University of Gävle
Department of occupational health science and psychology
Kungsbäcksvägen 47
801 76 Gävle
SWEDEN
sven.svensson@hig.se

[1] Department of occupational health science and psychology, University of Gävle, SWEDEN

[2] University of Gävle, Faculty of Education and Business Studies, Sweden

sven.svensson@hig.se
david.hallman@hig.se
svenderik.mathiassen@hig.se
marina.heiden@hig.se
arne.fagerstrom@hig.se
jean.claude.mutiganda@hig.se
gunnar.bergstrom@hig.se

Word count (excluding title page, abstract, references, figures and tables): 3507

Keywords: flexibility; non-standard employment; flexible work arrangements; economic sustainability; social sustainability; physical work environment; psychosocial work environment; health

For peer review only

FLexible work, Opportunity and Challenge (FLOC) for individual and organizational sustainability: Protocol for a prospective cohort study of non-standard employment and flexible work arrangements in Sweden

Abstract

Introduction: Flexibility in working life, including non-standard employment (NSE) and flexible work arrangements (FWA), offers the organization a better ability to adapt to changing conditions, while also posing considerable challenges, for organizations as well as workers. The aim of the Flexible work, Opportunity and Challenge (FLOC) study is to investigate associations at the organizational and individual levels between NSE and FWA, and economic and social sustainability. **Methods and analysis**: This prospective open cohort study will comprise approximately 10.000 workers 18-65 years in 6-8 public and private organizations in Sweden. We will use a comprehensive battery of measurement methods addressing financial viability, physical and psychosocial exposures, and physical and mental health; both at the organizational and the individual level. This includes valid survey questionnaires and register data, and, in subpopulations, technical measurements, interviews and diaries. Main exposures are type of employment and type of work arrangement. Main outcomes are indicators of organizational sustainability and, at the individual level, health and well-being. Data, collected over 54 months at approximately 18-months intervals, will be analyzed using multivariate methods considering main effects as well as potential effect modifiers. The analyses will take into account that respondents are nested in organizations, divisions and/or with specific managers.
Ethics and dissemination: FLOC is approved by the Swedish Ethical Review Authority (decision no. 2019-06220; 2020-06094; 2021-02725). Data will be published in peer-reviewed journals and presented at international conferences, and researchers will assist the organizations in improving policies and routines for employment and organization of work.

Article Summary

Strengths and limitations of this study

- Enrolling organizations in different sectors that employ workers with different socioeconomic status, ethnicity and gender, allows for addressing effect modification in sub-groups of employment conditions and work arrangements.
- Repeated measurements of exposures and outcomes allow for estimation of temporal relationships.
- FLOC will combine survey data, interviews and diaries with objective data gathered from company records, registers and technical measurements of physical workload and psychophysiological indicators. This will offer a comprehensive record of exposures and outcomes.
- Non-standard workers are only temporarily affiliated with a certain employer. This could result in a high dropout rate.

For peer review only - http://bmjopen.bmj.com/site/about/guidelines.xhtml

Introduction

Increased global competition and new Information and Communication Technology (ICT) has brought about an increased need as well as an improved capacity for flexibility in organizations [1]. Non-standard employment (NSE, the hiring of different sorts of temporary staff) [2] mainly provides flexibility for the organization, whereas flexible work arrangements (FWA, employment agreements allowing work to be performed at a discretionary time and place) mainly provide flexibility for the workers [3].

Both NSE and FWA could potentially benefit both employers and workers. However, this potential is not always realized; NSE and FWA can imply more autonomy but also that you will have to follow others' needs [4 5]. This is highly actualized during the ongoing COVID-19 pandemic where many organizations have been able to maintain operations and employments because of FWA. At the same time, non-standard workers (NSW) have in many cases been the first to be laid off [6]. Thus, understanding the tradeoff between positive and negative aspects of flexible work is a pertinent issue.

NSE and FWA can also lead to work environment and health challenges, hampering economic and social sustainability. Economic and social sustainability refers to the long-term balance between the needs of different stakeholders in organizations and companies. This includes organizations' need to maintain sound finances while, at the same time, provide a health promoting work environment [7 8], and even the needs of individuals to sustain health and well-being until retirement [8]. Results regarding organizational outcomes of NSE and FWA are rare and mixed. Some evidence suggests that industries with a large extent of NSE have a weaker product innovation propensity. This is a matter of current debate [9].

Work environment and health in non-standard employment

It may be difficult to involve NSW in occupational health and safety routines, for example due to ambiguities in the division of responsibilities[10] or communication difficulties[11] . NSE appears associated with physical and mental ill-health [12 13] and increased risk for occupational injury [14]. However, mixed findings have been reported [13-15]. NSE often results in poor physical and psychosocial work environment [12 16-19]. However, differences compared to standard work appears small [20].

Studies of NSE often suffer from methodological weaknesses [13-15 21]. Specific types of NSE appear to be differently associated with work environment and health, but most studies mix various forms of NSE [21]. Most studies are cross-sectional. Few studies have compared the working conditions of NSW to those of standard workers. Most previous studies have focused on the psychosocial rather than the physical work environment. Most often, non-validated instruments and self-reports have been used [12 15], likely introducing bias, e.g. when measuring physical activity [22].

Work environment and health in flexible work

Before COVID-19, 40 - 65% of the Swedish workforce could, to various extents, decide their working hours [23] or work from home [24]. The prevalence rates of telework (e.g. working from home) have increased since the outbreak of the COVID-19 pandemic [25 26]. Of those working from home in the summer of 2020 due to COVID-19, 46% did so for the first time [27].

FWA may improve work-life balance for workers, and potentially benefit productivity of organizations [28]. At the same time, employers may find it difficult to maintain occupational health and safety routines and a good psychosocial working environment for flexible workers [29-31]. Although FWA may allow workers a better work-life balance [32], it also puts higher demands on workers to set limits to their work [33 34]. Failure to set such limits may contribute to excessive workload and overtime work, insufficient time for recovery, and increased stress [35]. Most research on health in workers with FWA is cross-

For peer review only - http://bmjopen.bmj.com/site/about/guidelines.xhtml

sectional [28 32], and evidence of long-term health effects of FWA remains scarce [36]. Prospective studies are needed to improve knowledge, aiming at determining potential temporal associations between NSE and FWA on the one hand, and organizational viability, the work environment, and health and well-being of the workers, on the other.

Overall aim

The overall aim of the present 'FLexible work - Opportunities and Challenges' (FLOC) study is to determine in which ways, and to which extent NSE and FWA are associated with economic and social sustainability, in terms of the financial viability of organizations, their work environment, and the workers' health and well-being.

Primary aims and research questions

Aim 1: To evaluate the extent to which different forms of NSE and FWA are associated with the financial viability of the organization, its work environment, and health and well-being among the workers.

RQ1: What are the opportunities and challenges of NSE/FWA in the context of financial viability and management accounting and control?

RQ2: To which extent are different forms of NSE/FWA related to physical and psychosocial exposures at work?

RQ3: To which extent are different forms of NSE/FWA related to outcomes describing health and well-being?

RQ4: To which extent do the relationships determined in RQ1-RQ3 differ between occupational sectors, and according to age, gender and socioeconomic status of the worker?

Aim 2: To evaluate the contents and implementation of organizational policies and routines regarding NSE/FWA.

RQ5: What are the contents of organizational NSE/FWA policies and routines?

RQ6: How knowledgeable are managers about NSE/FWA-policies and routines?

RQ7: How do managers use/implement NSE/FWA-policies and routines in everyday work?

Methods and analysis

Design

FLOC is a prospective study with an open cohort approach, i.e. new participants can be added at different time points. The cohort combines surveys with register data, technical measurements, diaries and interviews. Measurements are performed at four time points, about 18 months apart. The overall design and timeline of the study are shown in Figures 1 and 2. The project is followed by a reference group with representatives from trade unions, the Confederation of Swedish Enterprise, the Swedish Work Environment Authority and universities.

For peer review only - http://bmjopen.bmj.com/site/about/guidelines.xhtml

Recruitment

We will apply convenience sampling of organizations and employees in organizations. Recruitment strategies include writing on a well-attended work environment blog, e-mailing potential organizations, and giving presentations for unions and employer organizations. The study population will consist of approximately 10 organizations within private and public sectors in Sweden.

Eligibility criteria

Recruitment of organizations will be concentrated to mid-Sweden. We strive to include organizations from both the private and public sector. Large organizations (>250) are prioritized. Eligible organizations allow us to register FWA for their workers, and/or they have a relatively large fraction of NSWs. In total, the participating organizations will employ approximately 6.000 blue-collar and white-collar workers. Eligible study participants are individuals 18-65 years old at baseline. Those employed because of political labor market initiatives at baseline are excluded (See figure 1). The organizations should be willing to share financial records as well as data on e.g. sick-leave, form of employment should be willing to allow measurements during working hours, and they should be willing to commit to the collaboration over several years.

FIGURE 1 HERE

Figure 1 Recruitment of study population and data collection (intended numbers)

Study procedures

Data will be collected from the approximately 6000 employees of the participating organizations between spring 2020 and autumn 2025. Data include excerpts from the Longitudinal integrated database for health insurance and labor market studies (LISA) at Statistics Sweden[37] , organizational data, a questionnaire to the workers, technical measurements of physical activities and heart rate, diaries and interviews. The questionnaire will be administered at baseline (T0), followed by three follow-ups at approximately 18-months intervals. Data will be combined with information from organizational records and the LISA-register over the 4.5 year follow-up period (T0 through T3). At each measurement point, participants will be asked for their informed consent to be followed in the LISA database and in organizational records via their social security number. Participants are also asked for their private contact information in case they leave the organization. This means that follow-up data will be available even for NSEs that have, for instance, changed employer, become unemployed, or turned to studies.

The FLOC questionnaire will mainly be composed of digital versions of validated questions from, e.g. COPSOQ III[38], the standardized questionnaire for the analysis of musculoskeletal symptoms[39], and the Karolinska sleep questionaire[40] (see table 1 for a complete list of questionnaires). It will be sent to all employees in the participating organizations, primarily via e-mail. The electronic FLOC questionnaire will be hosted by Qualtrics (Provo, Utah, USA). Depending on the accessibility of different groups of workers, the questionnaire will also be distributed via sms or in a paper-and-pencil version.

Data from organizational records will be handed over by the organizations HR-departments in excel spreadsheets before the measurements, completed for any new worker at each follow up. For

For peer review only - http://bmjopen.bmj.com/site/about/guidelines.xhtml

participants having given their consent to be followed in organizational records, the organizations will give complementing information regarding social security number, salary, and annual days of sick leave for each employee. The social security numbers obtained from the organizational records will also be used to follow each employee and obtain outcome variables from the LISA register during 2020-2024.

Indicators for calculating organizational financial viability will be given by each organization's accounts department and HR-department in excel spreadsheets during each data collection wave. Documents for policies and routines will be provided by the HR-departments.

In a sub-cohort, technical measurements of physical work load will be performed during work time and non-work time at T0 and T2 (details below). Participants will be recruited via an item in the questionnaire as well as via information given at notice boards in each organization. Measurement equipment will be administered at the workplace or sent to the participant's home address along with an extensive instruction for attachment and operation. In the latter instance, a trained instructor is available via the video conference tool Zoom.

Interviews with managers will be performed after the baseline measurement. Participants will be recruited via the HR departments at the organizations, and via e-mail. The interviews will be recorded and transcribed verbatim.

FIGURE 2 HERE

Figure 2 Flow of the data collection

Exposures, outcomes and contextual factors

Addressing the influence of NSE and FWA on economic and social sustainability at the organizational and individual level requires a number of exposures and outcomes to be followed. Also, a broad range of potential precursors and intermediate outcomes will be assessed to capture relevant aspects of physical and psychosocial exposures at work in different data sources. In addition, there is a need to consider characteristics and contextual factors specific to each organization. Table 1 shows a complete list of exposures, outcomes, precursors and contextual factors, with references to data sources.

Primary exposures

Main exposures are NSE and FWA. NSE refers, in the present study, to different kinds of temporary employment, and to temporary agency workers[2]. The main FWA will be telework, or combinations of telework and on-site work, so-called hybrid work [41]. Data on NSE and FWA will be collected in the questionnaire and from organizational records. Self-reported exposure data will be used when no organizational data are available. NSE is recorded using a self-developed item based on the forms of employment registered in Statistic Sweden's Labor Force Surveys [42]. A flexible work arrangement, according to the worker, is recorded using self-developed items for FWAs concerning its type, prevalence, duration and extent (days per week and total hours).

Primary outcomes

Organizational records provide information about the financial viability of organizations, including indicators for value added, financial ratios, wages, turnover, productivity, sick-leave costs and - conditional on participants' consent - individual sick-leave in days. The LISA-register will be used to

For peer review only - http://bmjopen.bmj.com/site/about/guidelines.xhtml

obtain additional outcome data of relevance for financial viability in the organizations for 2020-2024. Partial sick-leave days are transformed and standardized into full sick days (net days), wages will be analyzed in terms of net income from employment as well as total income, , and job turnover is measured as number of employers during the follow-up period. Reduced work performance at the individual level is measured by two items for productivity impairment, one due to ill-health and one due to work environment problems [43 44].

Self-rated sickness absence and presenteeism will be measured by one item each [45]. Individual semi-structured interviews with 15-20 managers will give information on how management perceives control in a context of NSE and FWA. The interviews will concern managers' experiences of leading working groups with NSE or FWA, and their ideas of challenges and opportunities in connection with hiring NSW or in connection with FWA. Examples of question areas are advantages and disadvantages for managerial control of the working environment, and production consequences of hiring NSW or in connection with FWA.

Worker health and well-being will be self-rated, measured with questionnaires for general health (short form health survey, SF-36) [46], musculoskeletal pain [39], exhaustion, depression and job satisfaction from Copenhagen psychosocial questionnaire III (COPSOQ III) [38], sleep-quality Karolinska Sleep Questionaire)[40], well-being (the world health organization's well-being index, WHO-5) [47], need for recovery (short form scale)[48], work ability (a single item from [49]), work-life balance (a validated single item) [50], work-life conflict [51], and a valid stress item [52].

Secondary exposures, secondary outcomes and contextual factors

Self-reported data regarding the physical and psychosocial work environment will include biomechanical load based on scales from the MUSIC-study [53], physical variation assessed with an item from COPSOQ III [38], physical activity measured with IPAQ [54], and physical exertion using a single item from Borg and Kaijser [55]. Additional self-developed items will be used to measure mental exertion and working postures. Psychosocial exposures (job demands, influence, social support, recognition, predictability, job insecurity, management quality, vertical trust, organizational justice, offensive treatment, demands for availability) is measured using indices or single items from COPSOQ III [38]. Psychosocial safety climate is measured with the 4-item psychosocial safety climate scale [56]. Self-developed questions address even functionality of IT-tools, overtime work, voluntariness regarding employment form and FWA, perceived demands for availability, perceived flexibility, occurrence of a policy for flexible work, commuting routines, and private financial expenses related to flexible work.

Objective data on physical exposures during work and non-work time will be collected by technical measurements, using small-size tri-axial accelerometers fixed to the thigh. Data will be collected over 7 days, and processed and analyzed using Acti4 [57].. We will quantify the occurrence and temporal pattern of sitting, standing and physical activities [58-60]. Data will be allocated to work and non-work time based on diaries [61]. Heart rate will be measured using a Bodyguard2 monitor with a standard 2-lead configuration, estimating general metabolic workload, e.g. in percent of heart rate reserve. Heart rate variability will be analyzed [62] and used as a marker of autonomic cardiac modulation.

Organizational records will give information on availability and content of policies for NSE and FWA. They will also be used to nest respondents in organizational divisions and to specific managers. Interviews with managers will give information on the knowledge and use of NSE/FWA policies.

Exposures

For peer review only - http://bmjopen.bmj.com/site/about/guidelines.xhtml

Type of employment contract	1; 2a
Duration of employment contract	1; 2a
Type of flexible work arrangement	1; 2a
Extent of flexible work	2a
Experience of flexible work	2a
Outcomes	
Financial viability	
Value added and ratios	1
Sick-leave	1; 2b; 3
Wages	1; 3
Turnover	1; 3
Productivity	1; 2c-d
Presenteeism	2b
Management control	4
Worker health and well-being	
General health	2e
Musculoskeletal pain	2f
Depression	2g
Well-being	2h
Job satisfaction	2g
Work-ability	2i
Exhaustion	2g
Stress	2j
Sleep -Quality	2k
Need for recovery	2l
Work-life balance	2m
Work-life conflict	2n
Intermediate outcomes, precursors and contextual factors	
Voluntariness, employment contract	2a
Voluntariness, flexible work	2a
Management, leadership quality	2g; 4
Job demands	2g
Influence	2g
Social support	2g
Recognition	2g
Predictability	2g
Job insecurity	2g
Vertical trust	2g
Organizational justice	2g
Offensive treatment	2g
Psychological safety climate	2o
Functionality, IT-tools	2a

For peer review only

Overtime work	2a
Perceived demands for availability	2g
Perceived flexibility	2a
Occurrence/content of NSE-policy	1
Occurrence/content of flexible work-policy	1
Private expenses related to FW	2a
Biomechanical load	2p
General metabolic load	5
Physical activities during work and non-work time	5
Physical variation	2g; 5
Physical exertion	2q; 5
Mental exertion	2a
Heart rate	5
General metabolic workload	5

1: Organizational records; 2a: Self-developed survey item; 2b: Survey[63]; 2c: Survey[43]; 2d: Survey[44]; 2e: Survey[46]; 2f: Survey[39]; 2g: Survey[38]; 2h: Survey[47]; 2i: Survey[49]; 2j: Survey[52]; 2k: Survey[40]; 2l: Survey[48]; 2m: Survey[50]; 2n: Survey[51]; 2o: Survey[56]; 2p: Survey[53]; 2q: Survey[55]; 3: LISA-register; 4: Interview; 5: Technical measurement

Table 1 Exposures, intermediate outcomes, and long-term outcomes

Confounders

To avoid the risk of mixing effects of a certain exposure with the effect of another variable, i.e. confounding[64], potential confounders will be identified from a large array of variables, for instance demographic and socioeconomic factors, union membership, smoking habits, physical activity during leisure, or prevalence of diagnosed disease. The selection of confounders will depend on the specific research questions in different studies, as well as theoretical and empirical considerations. Data for confounders will be collected from organizational records, the LISA-register and the questionnaire.

Sample size

The sample size was guided by a priori power analyses of primary exposures on the individual level (NSE/FWA) and outcomes (wellbeing, exhaustion, register information from organizational records on sickness absence in days). We estimated that about 400 workers per employment category (e.g. temporary contract vs standard employment) or work arrangements category (e.g. telework vs. on-site work) would be sufficient to detect significant ($p<0.05$) differences of 5 scale points with 80% power for wellbeing and exhaustion. The same sample size is also sufficient to detect a difference of 12 days sick leave between categories of workers. We aim at including 6.000 workers in the cohort. Out of these, 2000 will be NSW and 4000 will have FWAs. We expect a response rate of approximately 50% at baseline and a total drop out of, at the most, 30%. This will give enough respondents to adjust for relevant covariates and possible effect modification (e.g. by age and sex), in comparisons between categories of workers. Caution will be taken to limit the number of variables in the models, as allowed by the size of the dataset.

For peer review only - http://bmjopen.bmj.com/site/about/guidelines.xhtml

Data analysis

Workers with NSE will be compared with standard workers. Workers with FWA will be compared to those not having FWA. For FWA, duration and extent will be included as modifiers in the analyses. For both NSE and FWA, preference for NSE/FWA will be included as modifiers. Differences in outcomes over time will be analyzed primarily using Linear Mixed Models or Generalized Linear Mixed Models, depending on scale (continuous/categorical) and the distribution of data, considering main effects as well as potential modifiers. Respondents nested in different sectors, organizations and/or departments as well as to specific managers will be addressed specifically where possible. Groups may also be stratified based on gender, age and socioeconomic status. Theoretically relevant confounders will be considered in all analyses. Data loss will be inspected and treated using appropriate analytical imputation methods. Interviews will be analyszed using thematic analysis, e.g. qualitative content analysis [65].

Ethics and dissemination

The study is approved by the Swedish Ethical Review Authority (decisions no. 2019-06220, 2020-06094 and 2021-02725), and will be performed according to the declaration of Helsinki. FLOC-data will be published in peer-reviewed journals and presented at international conferences. Reports will be written to, and discussed with, participating organizations. Where relevant, researchers will assist the organizations in improvement of policies and routines. Results from the study will be continuously discussed with the cohort's reference group, which consists of members from employers' organizations and unions, representatives for the Swedish Work Environment Authority, and researchers. The FLOC-project group will be open for collaboration with other researchers and data will be shared, conditional on the provision of a research plan to the project group and congruence with the ethical approval.

The FLOC study is expected to contribute with original evidence concerning the effects of flexible employment forms and working arrangements. The study will initiate a new Swedish cohort on working conditions and health of workers with NSE and FWA. The study is based on a prospective approach with repeated measurements of exposures and outcomes, which allows for estimation of reliable risk estimates and minimizes bias due to reversed causation. To the best of our knowledge, this is the first study that investigates both the physical and psychosocial working environment and its associated effect on financial viability in the organization, and on health among the workers in an NSE and FWA context. The combination of register data, organizational records, questionnaire data, technical measurements and interviews will give a comprehensive contribution to research addressing NSE and FWA. The combined focus on different forms of NSE and FWA also facilitates increased understanding of how increased flexibility in working life affects companies and employees. Enrolling large representable organizations in different occupational sectors that employ workers with different socioeconomic status, ethnicity and gender allows for addressing effect modification by employment conditions, work arrangements and personal factors. At the same time, the heterogeneity of organizations is a limitation of the project since it leads to reduced opportunities to generalize the results to a specific sector or type of organizational context. Nevertheless, the comprehensive set of variables studied, obtained from a variety of data sources, will enable us to draw conclusions of relevance to today's working life. The development towards increased flexibility in working life opens up challenges and opportunities for employers and employees alike. We hope that the FLOC study will contribute to a more sustainable working life for both employers and employees.

For peer review only - http://bmjopen.bmj.com/site/about/guidelines.xhtml

For peer review only

Authors Contributions

GB, SEM, DH, MH, AF and SS conceptualized the idea. GB, SEM, DH, MH and SS conceived the study. GB, DH, MH and SEM developed the overall design. JCM contributed the section on economic and social sustainability. SS drafted the manuscript and all authors revised it critically. All authors have read and approved the manuscript in its present form.

Funding statement

This work was supported by FORTE (grant number 2019-01257) and AFA Insurance (grant number 200244).

Competing interests

None declared

For peer review only

References

1. Messenger J. Working time and the future of work. Geneva, Switzerland: ILO 2018.
2. Kalleberg AL. Nonstandard Employment Relations: Part-Time, Temporary and Contract Work. *Annual Review of Sociology* 2000;26:341-65.
3. Joyce K, Pabayo R, Critchley JA, et al. Flexible working conditions and their effects on employee health and wellbeing. *Cochrane Database Syst Rev* 2010;2
4. Isidorsson T, Kubisa J. Job quality in an era of flexibility : experiences in a European context. London: Routledge 2019.
5. MacEachen E, Polzer J, Clarke J. "You are free to set your own hours": Governing worker productivity and health through flexibility and resilience. *Social Science & Medicine* 2008;66(5):1019-33. doi: https://doi.org/10.1016/j.socscimed.2007.11.013
6. Spasova S, Ghailani D, Sabato S, et al. Non-standard Workers and the Self-employed in the EU: Social Protection During the Covid-19 Pandemic. *ETUI Research Paper-Report* 2021
7. Fagerström A, Hartwig F, Cunningham G. Accounting and Auditing of Sustainability: Sustainable Indicator Accounting (SIA). *Sustainability and Climate Change* 2017;10(1):45-52. doi: 10.1089/sus.2017.29080.af
8. Ajmal MM, Khan M, Hussain M, et al. Conceptualizing and incorporating social sustainability in the business world. *International Journal of Sustainable Development & World Ecology* 2018;25(4):327-39. doi: 10.1080/13504509.2017.1408714
9. Cetrulo A, Cirillo V, Guarascio D. Weaker jobs, weaker innovation. Exploring the temporary employment-product innovation nexus, 2018.
10. Gossett LM. Falling between the Cracks: Control and Communication Challenges of a Temporary Workforce. *Management Communication Quarterly* 2006;19(3):376-415. doi: 10.1177/0893318905280327
11. Quinlan M, Bohle P. Contingent work and occupational safety. In: Barling J, Frone MR, eds. The psychology of workplace safety. Washington, D.C.: American Psychological Association 2004.
12. Caban-Martinez AJ, Santiago KM, Stillman J, et al. Physical Exposures, Work Tasks, and OSHA-10 Training Among Temporary and Payroll Construction Workers. *Journal of occupational and environmental medicine* 2018;60(4):e159-e65.
13. Rönnblad T, Grönholm E, Jonsson J, et al. Precarious employment and mental health: a systematic review and meta-analysis of longitudinal studies. *Scandinavian Journal of Work, Environment & Health* 2019
14. Koranyi I, Jonsson J, Rönnblad T, et al. Precarious employment and occupational accidents and injuries–a systematic review. *Scandinavian journal of work, environment & health* 2018
15. Hünefeld L, Gerstenberg S, Hüffmeier J. Job satisfaction and mental health of temporary agency workers in Europe: a systematic review and research agenda. *Work & Stress* 2020;34(1):82-110. doi: 10.1080/02678373.2019.1567619
16. Roquelaure Y, LeManach AP, Ha C, et al. Working in temporary employment and exposure to musculoskeletal constraints. *Occupational medicine* 2012;62(7):514-18.
17. Bernhard-Oettel C, Sverke M, De Witte H. Comparing three alternative types of employment with permanent full-time work: How do employment contract and perceived job conditions relate to health complaints? *Work & Stress* 2005;19(4):301-18.
18. Wagenaar AF, Kompier MA, Houtman IL, et al. Employment contracts and health selection: unhealthy employees out and healthy employees in? *Journal of Occupational and Environmental Medicine* 2012;54(10):1192-200.
19. Lemanski S. Stress und gesundheit in der zeitarbeit. In: Bouncken RB, Bornewasser M, eds. Zeitarbeit und Flexibilisierung: Ergebnisse des Forschungsprojektes Flex4Work München: Hampp. 2012:75-107.

For peer review only - http://bmjopen.bmj.com/site/about/guidelines.xhtml

20. Bernhard-Oettel C, Leineweber C, Westerlund H. Staying in or switching between permanent, temporary and self-employment during 2008–2010: Associations with changing job characteristics and emotional exhaustion. 2019;40(2):215-37. doi: 10.1177/0143831x18804648
21. Imhof S, Andresen M. Unhappy with well-being research in the temporary work context: mapping review and research agenda. *The International Journal of Human Resource Management* 2018;29(1):127-64.
22. Gupta N, Heiden M, Mathiassen S, et al. Is self-reported time spent sedentary and in physical activity differentially biased by age, gender, body mass index, and low-back pain? *Scandinavian Journal of Work, Environment & Health* 2018(2):163-70. doi: 10.5271/sjweh.3693
23. Eurofound. European Quality of Life Survey 2016: Quality of life, quality of public services, and quality of society. In: Union POotE, ed. Luxembourg, 2017.
24. OECD BF. Be Flexible! Background brief on how workplace flexibility can help European employees to balance work and family: OECD Publishing, 2016.
25. EC. Telework in the EU before and after the COVID-19: where we were, where we head to. In: Comission E, ed.: European Comission, 2020.
26. Matthew D, Harley F, A. LM, et al. Ability to work from home: evidence from two surveys and implications for the labor market in the COVID-19 pandemic. *Monthly Labor Review* 2020;U.S. Bureau of labor statistics
27. Eurofound. Living, working and COVID-19,. In: Eurofound, ed. Luxembourg: Publications Office of the European Union, 2020.
28. Allen TD, Golden TD, Shockley KM. How effective is telecommuting? Assessing the status of our scientific findings. *Psychological Science in the Public Interest* 2015;16(2):40-68.
29. Cairns TD. What Will Tip the Scales for Flexible Work Arrangements—Motivation or Collaboration? *Employment Relations Today* 2013;40(2):29-33.
30. Messenger JC. Working anytime, anywhere: The evolution of Telework and its effects on the world of work. *IusLabor* 2017(3)
31. Tavits G. Tasks and Responsibilities of an Employer in a Digital Age: How to Comply with Applicable Requirements for Work Conditions. *Juridica Int'l* 2018;27:79.
32. Nijp HH, Beckers DGJ, Geurts SAE, et al. Systematic review on the association between employee worktime control and work-non-work balance, health and well-being, and job-related outcomes. *Scandinavian Journal of Work, Environment & Health* 2012;38(4):299-313.
33. Currie J, Eveline J. E-technology and work/life balance for academics with young children. *Higher Education* 2011;62(4):533-50.
34. Golden TD. The role of relationships in understanding telecommuter satisfaction. *Journal of Organizational Behavior* 2006;27(3):319-40.
35. Mellner C. After-hours availability expectations, work-related smartphone use during leisure, and psychological detachment. *International Journal of Workplace Health Management* 2016;9(2):146-64. doi: 10.1108/IJWHM-07-2015-0050
36. Nijp HH, Beckers DGJ, van de Voorde K, et al. Effects of new ways of working on work hours and work location, health and job-related outcomes. *Chronobiology International* 2016;33(6):604-18. doi: 10.3109/07420528.2016.1167731
37. Sweden S. Longitudinal integrated database for health insurance and labour market studies 2021 [Available from: https://scb.se/en/services/ordering-data-and-statistics/guidance-for-researchers-and-universities/vilka-mikrodata-finns/longitudinella-register/longitudinal-integrated-database-for-health-insurance-and-labour-market-studies-lisa/.
38. Berthelsen H, Westerlund H, Bergström G, et al. Validation of the Copenhagen Psychosocial Questionnaire Version III and establishment of benchmarks for psychosocial risk management in Sweden. *International journal of environmental research and public health* 2020;17(9):3179.

For peer review only - http://bmjopen.bmj.com/site/about/guidelines.xhtml

39. Kuorinka I, Jonsson B, Kilbom A, et al. Standardised Nordic questionnaires for the analysis of musculoskeletal symptoms. *Applied ergonomics* 1987;18(3):233-37.
40. Nordin M, Åkerstedt T, Nordin S. Psychometric evaluation and normative data for the Karolinska Sleep Questionnaire. *Sleep and Biological Rhythms* 2013;11(4):216-26.
41. Halford S. Hybrid workspace: re-spatialisations of work, organisation and management. 2005;20(1):19-33. doi: https://doi.org/10.1111/j.1468-005X.2005.00141.x
42. Sweden S. Labour Force Surveys 2021 [Available from: https://scb.se/en/finding-statistics/statistics-by-subject-area/labour-market/labour-force-surveys/labour-force-surveys-lfs/.
43. Aboagye E, Jensen I, Bergström G, et al. Validity and test–retest reliability of an at-work production loss instrument. *Occupational Medicine* 2016;66(5):377-82. doi: 10.1093/occmed/kqw021 %J Occupational Medicine
44. Lohela Karlsson M, Bergström G, Björklund C, et al. Measuring Production Loss due to Health and Work Environment Problems: Construct Validity and Implications. *Journal of Occupational and Environmental Medicine* 2013;55(12)
45. Aronsson G, Gustafsson K. Sickness Presenteeism: Prevalence, Attendance-Pressure Factors, and an Outline of a Model for Research. 2005;47(9):958-66. doi: 10.1097/01.jom.0000177219.75677.17
46. Sullivan M, Karlsson J. The Swedish SF-36 Health Survey III. Evaluation of Criterion-Based Validity: Results from Normative Population. *Journal of Clinical Epidemiology* 1998;51(11):1105-13. doi: 10.1016/S0895-4356(98)00102-4
47. Topp CW, Østergaard SD, Søndergaard S, et al. The WHO-5 Well-Being Index: a systematic review of the literature. *Psychotherapy and psychosomatics* 2015;84(3):167-76.
48. Stevens ML, Crowley P, Garde AH, et al. Validation of a short-form version of the Danish need for recovery scale against the full scale. *International journal of environmental research and public health* 2019;16(13):2334.
49. Ilmarinen J. The work ability index (WAI). *Occupational medicine* 2007;57(2):160-60.
50. Bjärntoft S, Hallman DM, Mathiassen SE, et al. Occupational and Individual Determinants of Work-life Balance among Office Workers with Flexible Work Arrangements. *Int J Environ Res Public Health* 2020;17(4) doi: 10.3390/ijerph17041418 [published Online First: 2020/02/27]
51. Carlson DS, Kacmar KM, Williams LJ. Construction and Initial Validation of a Multidimensional Measure of Work–Family Conflict. *Journal of Vocational Behavior* 2000;56(2):249-76. doi: https://doi.org/10.1006/jvbe.1999.1713
52. Arapovic-Johansson B, Wåhlin C, Kwak L, et al. Work-related stress assessed by a text message single-item stress question. 2017;67(8):601-08.
53. Wiktorin C, Vingård E, Mortimer M, et al. Interview versus questionnaire for assessing physical loads in the population-based MUSIC-Norrtälje study. *American journal of industrial medicine* 1999;35(5):441-55.
54. Hagströmer M, Oja P, Sjöström M. The International Physical Activity Questionnaire (IPAQ): a study of concurrent and construct validity. *Public Health Nutrition* 2006;9(6):755-62. doi: 10.1079/PHN2005898 [published Online First: 2007/01/02]
55. Borg E, Kaijser L. A comparison between three rating scales for perceived exertion and two different work tests. *Scandinavian journal of medicine & science in sports* 2006;16(1):57-69.
56. Berthelsen H, Muhonen T, Bergström G, et al. Benchmarks for Evidence-Based Risk Assessment with the Swedish Version of the 4-Item Psychosocial Safety Climate Scale. *International Journal of Environmental Research and Public Health* 2020;17(22):8675.
57. Stemland I, Ingebrigtsen J, Christiansen CS, et al. Validity of the Acti4 method for detection of physical activity types in free-living settings: comparison with video analysis AU - Stemland, Ingunn. *Ergonomics* 2015;58(6):953-65. doi: 10.1080/00140139.2014.998724
58. Heiden M, Zetterberg C, Mathiassen SE. Trunk and upper arm postures in paper mill work. *Applied Ergonomics* 2019;76:90-96. doi: https://doi.org/10.1016/j.apergo.2018.12.004

For peer review only - http://bmjopen.bmj.com/site/about/guidelines.xhtml

59. Hallman DM, Srinivasan D, Mathiassen SE. Short-and long-term reliability of heart rate variability indices during repetitive low-force work. *European journal of applied physiology* 2015;115(4):803-12.
60. Hallman DM, Gupta N, Heiden M, et al. Is prolonged sitting at work associated with the time course of neck–shoulder pain? A prospective study in Danish blue-collar workers. *BMJ open* 2016;6(11):e012689.
61. Hallman DM, Birk Jørgensen M, Holtermann A. On the health paradox of occupational and leisure-time physical activity using objective measurements: Effects on autonomic imbalance. *PLoS One* 2017;12(5):e0177042.
62. Laborde S, Mosley E, Thayer JF. Heart Rate Variability and Cardiac Vagal Tone in Psychophysiological Research – Recommendations for Experiment Planning, Data Analysis, and Data Reporting. *Frontiers in Psychology* 2017;8:213. doi: 10.3389/fpsyg.2017.00213
63. Aronsson G, Gustafsson K. Sickness presenteeism: prevalence, attendance-pressure factors, and an outline of a model for research. *Journal of occupational and environmental medicine* 2005;47(9):958-66.
64. Rothman KJ. Epidemiology : an introduction. New York, NY: Oxford University Press 2012.
65. Graneheim UH, Lundman BJNet. Qualitative content analysis in nursing research: concepts, procedures and measures to achieve trustworthiness. 2004;24(2):105-12.

For peer review only

For peer review only - http://bmjopen.bmj.com/site/about/guidelines.xhtml

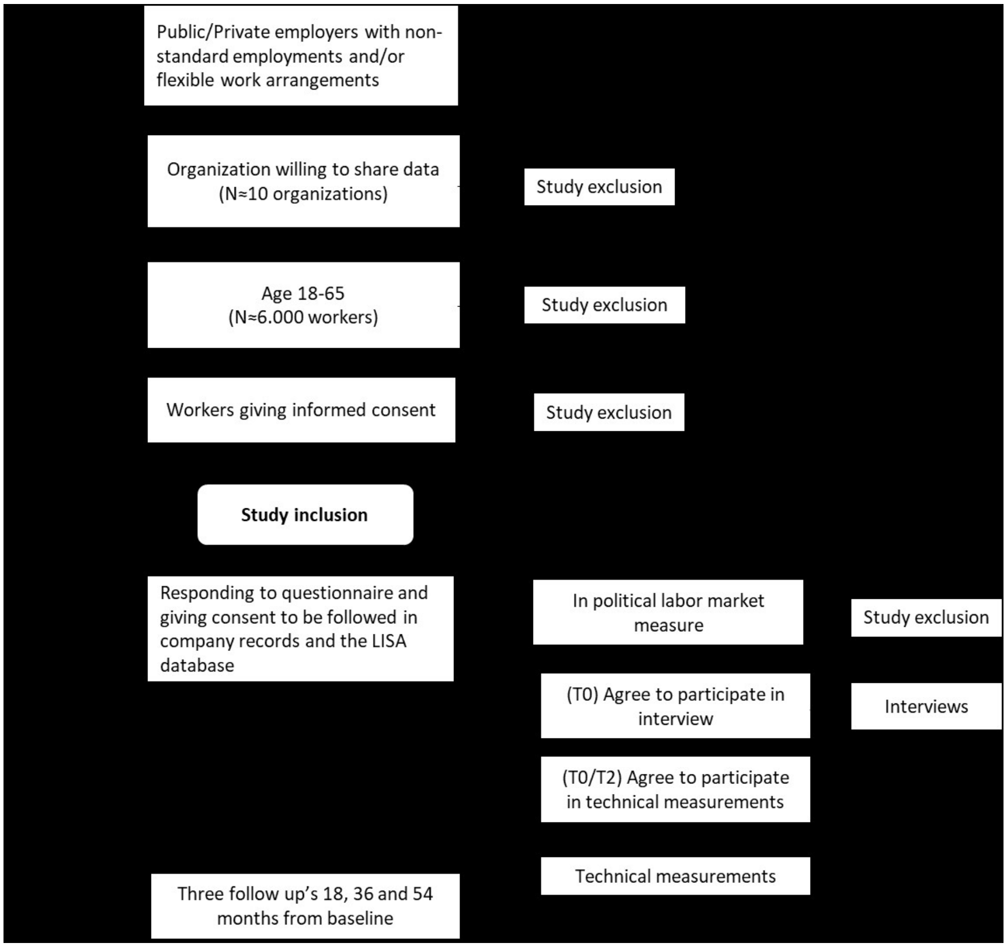

193x181mm (150 x 150 DPI)

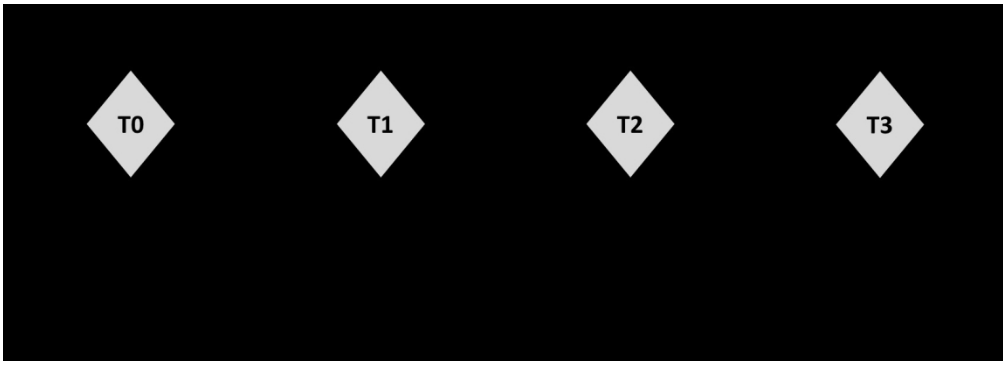

282x100mm (150 x 150 DPI)

STROBE 2007 (v4) Statement—Checklist of items that should be included in reports of *cohort studies*

Section/Topic	Item #	Recommendation	Reported on page #
**Title and abstract**	1	(*a*) Indicate the study's design with a commonly used term in the title or the abstract	1
		(*b*) Provide in the abstract an informative and balanced summary of what was done and what was found	1
**Introduction**			
Background/rationale	2	Explain the scientific background and rationale for the investigation being reported	2
Objectives	3	State specific objectives, including any prespecified hypotheses	3
**Methods**			
Study design	4	Present key elements of study design early in the paper	4
Setting	5	Describe the setting, locations, and relevant dates, including periods of recruitment, exposure, follow-up, and data collection	4-5
Participants	6	(*a*) Give the eligibility criteria, and the sources and methods of selection of participants. Describe methods of follow-up	5
		(*b*) For matched studies, give matching criteria and number of exposed and unexposed	n/a
Variables	7	Clearly define all outcomes, exposures, predictors, potential confounders, and effect modifiers. Give diagnostic criteria, if applicable	6-9
Data sources/ measurement	8*	For each variable of interest, give sources of data and details of methods of assessment (measurement). Describe comparability of assessment methods if there is more than one group	5-9
Bias	9	Describe any efforts to address potential sources of bias	9-10
Study size	10	Explain how the study size was arrived at	9
Quantitative variables	11	Explain how quantitative variables were handled in the analyses. If applicable, describe which groupings were chosen and why	n/a for protocol
Statistical methods	12	(*a*) Describe all statistical methods, including those used to control for confounding	n/a for protocol
		(*b*) Describe any methods used to examine subgroups and interactions	n/a for protocol
		(*c*) Explain how missing data were addressed	n/a for protocol
		(*d*) If applicable, explain how loss to follow-up was addressed	n/a for protocol
		(*e*) Describe any sensitivity analyses	n/a for protocol
**Results**			n/a for protocol

For peer review only

Participants	13*	(a) Report numbers of individuals at each stage of study—eg numbers potentially eligible, examined for eligibility, confirmed eligible, included in the study, completing follow-up, and analysed	n/a for protocol
		(b) Give reasons for non-participation at each stage	n/a for protocol
		(c) Consider use of a flow diagram	
Descriptive data	14*	(a) Give characteristics of study participants (eg demographic, clinical, social) and information on exposures and potential confounders	n/a for protocol
		(b) Indicate number of participants with missing data for each variable of interest	n/a for protocol
		(c) Summarise follow-up time (eg, average and total amount)	n/a for protocol
Outcome data	15*	Report numbers of outcome events or summary measures over time	n/a for protocol
Main results	16	(*a*) Give unadjusted estimates and, if applicable, confounder-adjusted estimates and their precision (eg, 95% confidence interval). Make clear which confounders were adjusted for and why they were included	n/a for protocol
		(*b*) Report category boundaries when continuous variables were categorized	n/a for protocol
		(*c*) If relevant, consider translating estimates of relative risk into absolute risk for a meaningful time period	n/a for protocol
Other analyses	17	Report other analyses done—eg analyses of subgroups and interactions, and sensitivity analyses	n/a for protocol
**Discussion**			
Key results	18	Summarise key results with reference to study objectives	10
**Limitations**			10
Interpretation	20	Give a cautious overall interpretation of results considering objectives, limitations, multiplicity of analyses, results from similar studies, and other relevant evidence	n/a for protocol
Generalisability	21	Discuss the generalisability (external validity) of the study results	n/a for protocol
**Other information**			
Funding	22	Give the source of funding and the role of the funders for the present study and, if applicable, for the original study on which the present article is based	11

*Give information separately for cases and controls in case-control studies and, if applicable, for exposed and unexposed groups in cohort and cross-sectional studies.

Note: An Explanation and Elaboration article discusses each checklist item and gives methodological background and published examples of transparent reporting. The STROBE checklist is best used in conjunction with this article (freely available on the Web sites of PLoS Medicine at http://www.plosmedicine.org/, Annals of Internal Medicine at http://www.annals.org/, and Epidemiology at http://www.epidem.com/). Information on the STROBE Initiative is available at www.strobe-statement.org.

For peer review only

BMJ Open

FLexible work: Opportunity and Challenge (FLOC) for individual, social and economic sustainability. Protocol for a prospective cohort study of non-standard employment and flexible work arrangements in Sweden

Journal:	*BMJ Open*
Manuscript ID	bmjopen-2021-057409.R1
Article Type:	Protocol
Date Submitted by the Author:	11-Apr-2022
Complete List of Authors:	Svensson, Sven; University of Gävle, Department of occupational health science and psychology<br>Hallman, David; University of Gävle, Department of occupational health science and psychology<br>Mathiassen, Svend ; University of Gävle, Department of occupational health science and psychology<br>Heiden, Marina; University of Gävle, Department of occupational health science and psychology<br>Fagerström, Arne; University of Gävle, Faculty of Education and Business Studies<br>Mutiganda, Jean Claude; University of Gävle, Faculty of Education and Business Studies<br>Bergström, Gunnar; University of Gävle, Department of occupational health science and psychology; Karolinska Institutet, Unit of Intervention and Implementation Research for Worker Health, Institute of Environmental Medicine
**Primary Subject Heading**:	Occupational and environmental medicine
Secondary Subject Heading:	Occupational and environmental medicine
Keywords:	EPIDEMIOLOGY, OCCUPATIONAL & INDUSTRIAL MEDICINE, SOCIAL MEDICINE

SCHOLARONE™
Manuscripts

FLexible work: Opportunity and Challenge (FLOC) for individual, social and economic sustainability. Protocol for a prospective cohort study of non-standard employment and flexible work arrangements in Sweden

Sven Svensson*[1], David Hallman[1], SvendErik Mathiassen[1], Marina Heiden[1], Arne Fagerström[2], Jean Claude Mutiganda[2], Gunnar Bergström[1, 3]

* Corresponding author:

Sven Svensson
University of Gävle
Department of occupational health science and psychology
Kungsbäcksvägen 47
801 76 Gävle
SWEDEN
sven.svensson@hig.se

[1] Department of occupational health science and psychology, University of Gävle, Sweden

[2] University of Gävle, Faculty of Education and Business Studies, University of Gävle, Sweden

[3] Unit of Intervention and Implementation Research for Worker Health, Institute of Environmental Medicine, Karolinska Institutet, Sweden

sven.svensson@hig.se
david.hallman@hig.se
svenderik.mathiassen@hig.se
marina.heiden@hig.se
arne.fagerstrom@hig.se
jean.claude.mutiganda@hig.se
gunnar.bergstrom@hig.se

Word count (excluding title page, abstract, references, figures and tables): 3845

Keywords: flexibility; non-standard employment; flexible work arrangements; economic sustainability; social sustainability; physical work environment; psychosocial work environment; health

For peer review only

FLexible work: Opportunity and Challenge (FLOC) for individual, social and economic sustainability. Protocol for a prospective cohort study of non-standard employment and flexible work arrangements in Sweden

Abstract

Introduction: Flexibility in working life, including non-standard employment (NSE) and flexible work arrangements (FWA), offers the organization a better ability to adapt to changing conditions, while also posing considerable challenges, for organizations as well as workers. The aim of the Flexible work, Opportunity and Challenge (FLOC) study is to investigate associations between NSE and FWA on the one hand, and individual, social and economic sustainability on the other. **Methods and analysis**: This prospective open cohort study targets approximately 8,000 workers 18-65 years in 8-10 public and private organizations in Sweden. We will use a comprehensive battery of measurement methods addressing financial performance, physical and psychosocial exposures, and physical and mental health; both at the organizational and the individual level. Methods includes valid survey questionnaires and register data, and, in subpopulations, technical measurements, interviews and diaries. Main exposures are type of employment and type of work arrangement. Main outcomes are indicators of social and economic sustainability and, at the individual level, health and well-being. Data, collected over 54 months at approximately 18-months intervals, will be analyzed using multivariate methods considering main effects as well as potential effect modifiers. The analyses will take into account that respondents are nested in organizations, divisions and/or have specific managers.

Ethics and dissemination: FLOC is approved by the Swedish Ethical Review Authority (decision no. 2019-06220; 2020-06094; 2021-02725). Data will be published in peer-reviewed journals and presented at international conferences, and researchers will assist the organizations in improving policies and routines for employment and organization of work.

Article Summary

Strengths and limitations of this study

- Recruiting organizations in different sectors employing workers with different socioeconomic status, ethnicity and gender, allows for addressing effect modification in sub-groups with varying employment conditions and work arrangements.
- Repeated measurements of exposures and outcomes allow for estimation of temporal relationships.
- FLOC will combine survey data, interviews and diaries with objective data collected from company records, registers and technical measurements of physical workload and psychophysiological indicators. This will offer a comprehensive array of exposures and outcomes.
- Non-standard workers are only temporarily affiliated with a certain employer. This could result in a high dropout rate.

For peer review only - http://bmjopen.bmj.com/site/about/guidelines.xhtml

Introduction

Increased global competition and new Information and Communication Technology (ICT) has brought about an increased need as well as an improved capacity for flexibility in organizations [1]. Non-standard employment (NSE, the hiring of different sorts of temporary staff) [2] mainly provides flexibility for the organization, whereas flexible work arrangements (FWA, employment agreements allowing work to be performed at a discretionary time and place) mainly provide flexibility for the workers [3].

Both NSE and FWA could potentially benefit both employers and workers. However, this potential is not always realized; NSE and FWA can imply more autonomy but also that you will have to follow others' needs [4 5]. This is highly actualized during the ongoing COVID-19 pandemic where many organizations have been able to maintain operations and employments because of FWA. At the same time, non-standard workers (NSW) have in many cases been the first to be laid off [6]. Thus, understanding the tradeoff between positive and negative aspects of flexibility in working life is a pertinent issue.

NSE and FWA may also represent challenges regarding work environment and health challenges that can hamper social and economic sustainability. Social and economic sustainability refers to the long-term balance between the needs of different stakeholders in organizations and companies. This includes organizations' need to maintain sound finances while, at the same time, providing a health promoting work environment [7 8], and even securing the needs of individuals for sustainable health and well-being until retirement [8].

Results regarding organizational effects of NSE and FWA are rare and mixed. Some evidence suggests that industries with a large extent of NSE have a weaker product innovation propensity[9], and that NSE is associated with either higher or lower wage costs[10]. Both issues are matters of current debates [9 10]. Literature addressing effects of hiring NSW on the economic performance of an organization is sparse, although low as well as high proportions of NSE might be negatively associated with financial performance [11].

Work environment and health in non-standard employment

It may be difficult to involve NSW in occupational health and safety routines, for example due to ambiguities in the division of responsibilities[12] or communication difficulties[13] . NSE appears associated with physical and mental ill-health [14-16] and increased risk for occupational injury [17]. However, mixed findings have been reported [15 17 18]. NSE often results in poor physical and psychosocial work environments [14 19-22]. However, differences compared to standard work appear small [23].

Studies of NSE often suffer from methodological weaknesses [15 17 18 24]. Specific types of NSE appear to be differently associated with work environment and health, but most studies mix various forms of NSE [24]. Most studies are cross-sectional. Few studies have compared the working conditions of NSW to those of standard workers. Most previous studies have focused on the psychosocial rather than the physical work environment. Most often, non-validated instruments and self-reports have been used [14 18], likely introducing bias, e.g. when measuring physical activity [25].

Work environment and health in flexible work

Before COVID-19, 40 - 65% of the Swedish workforce could, to various extents, decide their working hours [26] or work from home [27]. The prevalence rates of telework (e.g. working from home) have increased since the outbreak of the COVID-19 pandemic [28 29]. Of those working from home in the summer of 2020 due to COVID-19, 46% did so for the first time [30].

For peer review only - http://bmjopen.bmj.com/site/about/guidelines.xhtml

FWA may improve work-life balance for workers, and potentially benefit the productivity of organizations [31]. At the same time, employers may find it difficult to maintain occupational health and safety routines and a good psychosocial working environment for flexible workers [32-34]. The proportion of FWA in an organization may influence the working conditions even for workers with non-FWA[35]. Although FWA may allow workers a better work-life balance [36], it also puts higher demands on workers to set limits to their work [37 38]. Failure to set such limits may contribute to excessive workload and overtime work, insufficient time for recovery, and increased stress [39]. Most research on health in workers with FWA is cross-sectional [31 36], and evidence of long-term health effects of FWA remains scarce [40]. Prospective studies are needed to improve knowledge, aiming at determining potential temporal associations between NSE and FWA on the one hand, and individual, social and economic sustainability on the other [41-44].

Overall aim

The overall aim of the present 'FLexible work - Opportunities and Challenges' (FLOC) study is to determine in which ways, and to which extent NSE and FWA are associated with social and economic sustainability, in terms of the financial performance of organizations, their work environment, and workers' health and well-being.

Primary aims and research questions

Aim 1: To evaluate the extent to which different forms of NSE and FWA are associated with the financial performance of the organization, its work environment, and health and well-being among the workers.

RQ1: What are the opportunities and challenges presented by NSE/FWA in the context of an organization's financial performance and its management accounting and control?

RQ2: To which extent are different forms of NSE/FWA related to physical and psychosocial exposures at work?

RQ3: To which extent are different forms of NSE/FWA related to outcomes describing health and well-being?

RQ4: To which extent do the answers to RQ1-RQ3 differ between occupational sectors, and according to age, gender and socioeconomic status of the worker?

Aim 2: To evaluate the contents and implementation of organizational policies and routines regarding NSE/FWA.

RQ5: What are the contents of organizational NSE/FWA policies and routines?

RQ6: How knowledgeable are managers, workers and union representatives regarding policies and routines for NSE and FWA?

RQ7: How do managers use/implement policies and routines for NSE and FWA in everyday work?

For peer review only - http://bmjopen.bmj.com/site/about/guidelines.xhtml

Methods and analysis

Design

FLOC is a prospective study with an open cohort approach, i.e. new participants can be added at different time points. The cohort combines surveys with register data, technical measurements, diaries and interviews. Measurements are performed at four time points, about 18 months apart. The overall design and timeline of the study are shown in Figures 1 and 2. The project is followed by a reference group with representatives from trade unions, the Confederation of Swedish Enterprise, the Swedish Work Environment Authority and universities.

Recruitment

We will apply a purposive convenience sampling of organizations and employees within organizations. Recruitment strategies include distributing messages on a well-attended work environment blog, e-mailing potential organizations, and giving presentations for unions and employer organizations. The study population will, eventually, consist of 8-10 organizations within the private and public sectors in Sweden, representative of specific types and extents of NSE and/or FWA.Eligibility criteria

Recruitment of organizations will be concentrated to mid-Sweden. We strive to include organizations from both the private and public sector. Large organizations (>250) are prioritized. Eligible organizations will allow us to approach all employees, register FWA for all participating workers and/or have a relatively large fraction of NSWs. In total, the participating organizations will employ approximately 8,000 blue-collar and white-collar workers. Eligible study participants are individuals 18-65 years old at baseline. Those employed because of political labor market initiatives at baseline are excluded (See figure 1). The organizations should be willing to share financial records as well as data on e.g. sick-leave, form of employment, should be willing to allow measurements during working hours, and they should be willing to commit to the collaboration over several years.

FIGURE 1 HERE

Figure 1 Recruitment of study population and data collection (intended numbers)

Study procedures

Data will be collected from approximately 8,000 employees in the participating organizations between 2020-05-25 and 2025-11-30. Data include excerpts from the Longitudinal integrated database for health insurance and labor market studies (LISA) at Statistics Sweden[45], organizational data, a questionnaire to the workers, technical measurements of physical activities and heart rate, diaries and interviews. The questionnaire will be administered at baseline (T0), followed by three follow-ups at approximately 18-months intervals. Data will be combined with information from organizational records and the LISA-register over the 4.5 year follow-up period (T0 through T3). At each measurement point, participants will be asked for their informed consent to participate and be followed via their social security number in the LISA database and in organizational records. Thus, follow-up data will be available even for study participants that drop out of the study during the data collection period. Participants will also be asked for their private contact information which allows follow-up data to be

For peer review only - http://bmjopen.bmj.com/site/about/guidelines.xhtml

collected from study participants that have, for instance, changed employer, become unemployed, or turned to studies.

The FLOC questionnaire will mainly be composed of digital versions of validated questions from, e.g. COPSOQ III[46], the standardized questionnaire for the analysis of musculoskeletal symptoms[47], and the Karolinska sleep questionaire[48] (see table 1 for a complete list of questionnaires). It will be sent to all employees in the participating organizations, primarily via e-mail. The electronic FLOC questionnaire will be hosted by Qualtrics (Provo, Utah, USA), with survey data stored at servers in the EU. Depending on the accessibility of different groups of workers, the questionnaire will also be distributed via sms or in a paper-and-pencil version.

Data from organizational records regarding e.g. types of employment or employment rates will be handed over by the organizations HR-departments in excel spreadsheets before the measurements, completed for any new worker at each follow up. For participants having given their consent to be followed in organizational records, the organizations will give complementing information regarding social security number, salary, and annual days of sick leave for each employee. Salary and sick-leave data will be updated on a yearly basis. The social security numbers obtained from the organizational records will also be used to follow each employee and obtain outcome variables from the LISA register during 2020-2024.

Indicators for calculating organizational financial performance will be given by each organization's accounts department and HR-department in excel spreadsheets during each data collection wave. This data will include information on organizational structure, including which employees are subordinate to specific managers in each organizational department. Documents for policies and routines will be provided by the HR-departments.

In a sub-cohort, technical measurements of physical work load will be performed during work time and non-work time at T0 and T2 (details below). Participants will be recruited via an item in the questionnaire as well as via information given on notice boards in each organization. Measurement equipment will be administered at the workplace or sent to the participant's home address along with an extensive instruction for attachment and operation. In the latter instance, a trained instructor is available via the video conference tool Zoom.

Interviews with managers will be performed after the baseline measurement. Participants will be recruited via the HR departments at the organizations, and via e-mail. The interviews will be recorded and transcribed verbatim.

In some cases, we will have access to two data sources, e.g. as regards organizational policies. This allows us to examine possible differences between the two data sets and discuss these differences and their interpretation with relevant parties.

FIGURE 2 HERE

Figure 2 Flow of the data collection

For peer review only - http://bmjopen.bmj.com/site/about/guidelines.xhtml

Exposures, outcomes and contextual factors

Addressing the influence of NSE and FWA on social and economic sustainability at the organizational level and health and well-being at the individual level requires a number of exposures and outcomes to be followed. Also, a broad range of potential precursors and intermediate outcomes will be assessed to capture relevant aspects of physical and psychosocial exposures at work in different data sources. In addition, there is a need to consider characteristics and contextual factors specific to each organization. Table 1 shows a complete list of exposures, outcomes, precursors and contextual factors, with references to data sources.

Primary exposures

Main exposures are NSE and FWA. NSE refers, in the present study, to different kinds of temporary employment, and to temporary agency workers[2]. The main FWA will be telework, or combinations of telework and on-site work, so-called hybrid work [49]. Data on NSE and FWA will be collected in the questionnaire and from organizational records. Self-reported exposure data will be used when no organizational data are available. NSE is recorded using a self-developed item based on the forms of employment registered in Statistic Sweden's Labor Force Surveys [50]. A flexible work arrangement, according to the worker, is recorded using self-developed items for FWAs concerning its type, prevalence, duration and extent (days per week and total hours).

Primary outcomes

Organizational records provide information about the financial performance of organizations, including indicators for value added, financial ratios, wages, turnover, productivity, sick-leave costs and - conditional on participants' consent - individual sick-leave in days. The LISA-register will be used to obtain additional outcome data of relevance for financial performance in the organizations for 2020-2024. Partial sick-leave days are transformed and standardized into full sick-leave days (net days), wages will be analyzed at the individual level in terms of net incomes from employment after income tax as well as gross income before tax, and job turnover is measured as number of employers during the follow-up period. Work performance at the individual level is measured by two items for productivity impairment, one due to ill-health and one due to work environment problems [51 52].

Self-rated sickness absence and presenteeism will be measured by one item each [53]. Individual semi-structured interviews with 15-20 managers will give information on how management perceives control in the context of NSE and FWA. The interviews will concern managers' experiences of leading working groups with NSE or FWA, and their ideas of challenges and opportunities in connection with hiring NSW or in connection with FWA. Examples of question areas are advantages and disadvantages for managerial control of the working environment, and production consequences of hiring NSW or in connection with FWA.

Worker health and well-being will be self-rated, measured with questionnaires for general health (short form health survey, SF-36) [54], musculoskeletal pain [47], exhaustion, depression and job satisfaction from Copenhagen psychosocial questionnaire III (COPSOQ III) [46], sleep-quality (Karolinska Sleep Questionaire)[48], well-being (the World Health Organization's well-being index, WHO-5) [55], need for recovery (short form scale)[56], workability (a single item from [57]), work-life balance (a validated single item) [58], work-life conflict [59], and a valid stress item [60].

Secondary exposures, secondary outcomes and contextual factors

Information on working time is obtained through organizational records and in the questionnaire. Self-reported data regarding the physical and psychosocial work environment will include biomechanical load based on scales from the MUSIC-study [61], physical variation assessed with an item from COPSOQ

For peer review only - http://bmjopen.bmj.com/site/about/guidelines.xhtml

III [46], physical activity measured with IPAQ [62], and physical exertion using a single item from Borg and Kaijser [63]. Additional self-developed items will be used to measure mental exertion and working postures. Psychosocial exposures (job demands, influence, social support, recognition, predictability, job insecurity, management quality, vertical trust, organizational justice, offensive treatment, demands for availability) is measured using indices or single items from COPSOQ III [46]. Psychosocial safety climate is measured with the 4-item psychosocial safety climate scale [64]. Self-developed questions address even functionality of IT-tools, overtime work, voluntariness regarding employment form and FWA, perceived demands for availability, perceived flexibility, occurrence of a policy for flexible work, commuting routines, and private financial expenses related to flexible work.

Objective data on physical exposures during work and non-work time will be collected by technical measurements, using small-size tri-axial accelerometers fixed to the thigh. Data will be collected over 7 days, and processed and analyzed using Acti4 [65]. We will quantify the occurrence and temporal pattern of sitting, standing and physical activities [66-68]. Data will be allocated to work and non-work time based on diaries [69]. Heart rate will be measured using a Bodyguard2 monitor with a standard 2-lead configuration, estimating general metabolic workload, e.g. in percent of heart rate reserve. Heart rate variability will be analyzed [70] and used as a marker of autonomic cardiac modulation.

Organizational records will give information on availability and content of policies for NSE and FWA. They will also be used to nest respondents in organizational divisions and to specific managers. Interviews with managers, workers and union representatives will offer information on the knowledge of NSE/FWA policies and of the extent to which they are implemented.

For peer review only

For peer review only - http://bmjopen.bmj.com/site/about/guidelines.xhtml

Primary exposures	
Type of employment contract	1; 2a
Duration of employment contract	1; 2a
Type of flexible work arrangement	1; 2a
Extent of flexible work	2a
Experience of flexible work	2a
Primary outcomes	
Financial performance	
Value added and ratios	1
Sick-leave	1; 2b; 3
Wages	1; 3
Turnover	1; 3
Productivity	1; 2c-d
Presenteeism	2b
Management control	4
Worker health and well-being	
General health	2e
Musculoskeletal pain	2f
Depression	2g
Well-being	2h
Job satisfaction	2g
Work-ability	2i
Exhaustion	2g
Stress	2j
Sleep Quality	2k
Need for recovery	2l
Work-life balance	2m
Work-life conflict	2n
Secondary exposures, secondary outcomes and contextual factors	
Voluntariness, employment contract	2a
Voluntariness, flexible work	2a
Management, leadership quality	2g; 4
Job demands	2g
Influence	2g
Social support	2g
Recognition	2g
Predictability	2g
Job insecurity	2g
Vertical trust	2g
Organizational justice	2g
Offensive treatment	2g
Psychological safety climate	2o

For peer review only

For peer review only - http://bmjopen.bmj.com/site/about/guidelines.xhtml

Functionality, IT-tools	2a
Overtime work	2a
Perceived demands for availability	2g
Perceived flexibility	2a
Occurrence/content of NSE-policy	1
Occurrence/content of flexible work-policy	1
Private expenses related to FW	2a
Biomechanical load	2p
General metabolic load	5
Physical activities during work and non-work time	5
Physical variation	2g; 5
Physical exertion	2q
Mental exertion	2a
Heart rate	5
General metabolic workload	5

1: Organizational records; 2a: Self-developed survey item; 2b: Survey[71]; 2c: Survey[51]; 2d: Survey[52]; 2e: Survey[54]; 2f: Survey[47]; 2g: Survey[46]; 2h: Survey[55]; 2i: Survey[57]; 2j: Survey[60]; 2k: Survey[48]; 2l: Survey[56]; 2m: Survey[58]; 2n: Survey[59]; 2o: Survey[64]; 2p: Survey[61]; 2q: Survey[63]; 3: LISA-register; 4: Interview; 5: Technical measurement

Table 1 Exposures, outcomes and contextual factors

For peer review only

Confounders

To avoid the risk of mixing effects of a certain exposure with the effect of another variable, i.e. confounding[72], potential confounders will be identified from a large array of variables, for instance demographic and socioeconomic factors (including family situation), union membership, smoking habits, physical activity during leisure, or prevalence of diagnosed disease. The selection of confounders will depend on the specific research questions in different studies, as well as theoretical and empirical considerations. Data on confounders will be collected from organizational records, the LISA-register and the questionnaire. Data regarding the economic situation of the local community is available from Statistics Sweden.

Sample size

The sample size was guided by a priori power analyses of primary exposures on the individual level (NSE/FWA) and outcomes (wellbeing, exhaustion, register information from organizational records on sickness absence in days). For minimally important score differences of 0.5 SD, we estimated that about 200 workers per employment category (e.g. temporary contract and standard employment) or work arrangements category (e.g. telework and on-site work) would be sufficient to detect significant ($p<0.05$) differences of 10 scale points with 80% power for wellbeing and exhaustion and 12 scale points for burnout[46]. A similar sample size is also sufficient to detect a difference of 20 days sick leave between categories of workers. We aim at a target population of approximately 8,000 workers in the cohort. Out of these, approximately 4,000 will be standard workers, 2,000 NSW and 2,000 will have FWAs. In some cases, a particular worker may be represented in both the latter categories. We expect a response rate of approximately 50% at baseline and a total drop out of, at the most, 30%, which will

For peer review only - http://bmjopen.bmj.com/site/about/guidelines.xhtml

give approximately 2,800 workers in the final sample. This will give enough respondents to adjust for relevant covariates and possible effect modification (e.g. by age and sex), in comparisons between categories of workers. Caution will be taken to limit the number of variables in the models, as allowed by the size of the dataset.

Data analysis

Workers with NSE will be compared with standard workers. Workers with FWA will be compared to those not having FWA. For FWA, duration and extent will be included as modifiers in the analyses. For both NSE and FWA, preference for NSE/FWA will be included as modifiers. Differences in outcomes over time will be analyzed primarily using Linear Mixed Models or Generalized Linear Mixed Models, depending on scale (continuous/categorical) and the distribution of data, considering main effects as well as potential modifiers. Respondents nested in different sectors, organizations and/or departments as well as to specific managers will be addressed specifically where possible. Groups may also be stratified based on gender, age and socioeconomic status. Theoretically relevant confounders will be considered in all analyses. Data loss will be inspected and treated using appropriate analytical imputation methods. Interviews will be analyzed using thematic analysis, e.g. qualitative content analysis [73].

Patient and public involvement

No patients involved.

Ethics and dissemination

The study is approved by the Swedish Ethical Review Authority (decisions no. 2019-06220, 2020-06094 and 2021-02725), and will be performed according to the declaration of Helsinki. Participants answering the questionnaire will provide their informed consent in the on-line questionnaire. Participants in interviews and/or technical measurements will give their written informed consent to the researcher before starting the interview and/or starting measurements. FLOC-data will be published in peer-reviewed journals and presented at international conferences. Participating organizations will not receive information that can be tracked down to individual workers. Reports of results at an aggregated level will be delivered to, and discussed with, participating organizations. Based on these discussions, researchers will assist the organizations in improvement of policies and routines, where relevant. The researchers will not receive any economic compensation from any participating organization. Results from the study will be continuously discussed with the cohort's reference group, which consists of members from employers' organizations and unions, representatives for the Swedish Work Environment Authority, and researchers. The FLOC-project group will be open for collaboration with other researchers and data will be shared, conditional on the provision of a research plan to the project group and congruence with the ethical approval.

The FLOC study is expected to contribute with original evidence concerning the effects of flexible employment forms and working arrangements. The study will initiate a new Swedish cohort on working conditions and health of workers with NSE and FWA. The study is based on a prospective approach with repeated measurements of exposures and outcomes, which allows for estimation of reliable risk estimates and minimizes bias due to reversed causation. To the best of our knowledge, this is the first study that investigates both the physical and psychosocial working environment and its associated effect on financial performance in the organization, and on health among the workers in an NSE and FWA context. The combination of register data, organizational records, questionnaire data, technical measurements and interviews will give a comprehensive contribution to research addressing NSE and FWA. The combined focus on different forms of NSE and FWA also facilitates increased

For peer review only - http://bmjopen.bmj.com/site/about/guidelines.xhtml

understanding of how increased flexibility in working life affects companies and employees. Enrolling large representable organizations in different occupational sectors that employ workers with different socioeconomic status, ethnicity and gender allows for addressing effect modification by employment conditions, work arrangements and personal factors. At the same time, the heterogeneity of organizations is a limitation of the project since it leads to reduced opportunities to generalize the results to a specific sector or type of organizational context. Nevertheless, the comprehensive set of variables studied, obtained from a variety of data sources, will enable us to draw conclusions of relevance to today's working life. The development towards increased flexibility in working life opens up challenges and opportunities for employers and employees alike. We hope that the FLOC study will contribute to a more sustainable working life for both employers and employees.

Authors Contributions

GB, SEM, DH, MH, AF and SS conceptualized the idea. GB, SEM, DH, MH and SS conceived the study. GB, DH, MH and SEM developed the overall design. JCM contributed to the section on economic and social sustainability. SS drafted the manuscript and all authors revised it critically. All authors have read and approved the manuscript in its present form.

Funding statement

This work was supported by FORTE (grant number 2019-01257) and AFA Insurance (grant number 200244).

Competing interests

None declared

For peer review only

References

1. Messenger J. Working time and the future of work. Geneva, Switzerland: ILO 2018.
2. Kalleberg AL. Nonstandard Employment Relations: Part-Time, Temporary and Contract Work. *Annual Review of Sociology* 2000;26:341-65.
3. Joyce K, Pabayo R, Critchley JA, et al. Flexible working conditions and their effects on employee health and wellbeing. *Cochrane Database Syst Rev* 2010;2
4. Isidorsson T, Kubisa J. Job quality in an era of flexibility : experiences in a European context. London: Routledge 2019.
5. MacEachen E, Polzer J, Clarke J. "You are free to set your own hours": Governing worker productivity and health through flexibility and resilience. *Social Science & Medicine* 2008;66(5):1019-33. doi: https://doi.org/10.1016/j.socscimed.2007.11.013
6. Spasova S, Ghailani D, Sabato S, et al. Non-standard Workers and the Self-employed in the EU: Social Protection During the Covid-19 Pandemic. *ETUI Research Paper-Report* 2021
7. Fagerström A, Hartwig F, Cunningham G. Accounting and Auditing of Sustainability: Sustainable Indicator Accounting (SIA). *Sustainability and Climate Change* 2017;10(1):45-52. doi: 10.1089/sus.2017.29080.af
8. Ajmal MM, Khan M, Hussain M, et al. Conceptualizing and incorporating social sustainability in the business world. *International Journal of Sustainable Development & World Ecology* 2018;25(4):327-39. doi: 10.1080/13504509.2017.1408714
9. Reljic J, Cetrulo A, Cirillo V, et al. Non-standard work and innovation: evidence from European industries. *Economics of Innovation and New Technology* 2021 doi: 10.1080/10438599.2021.1893139
10. Albanese A, Gallo G. Buy flexible, pay more: The role of temporary contracts on wage inequality. *Labour Economics* 2020;64:101814. doi: https://doi.org/10.1016/j.labeco.2020.101814
11. Qian X, Wang H. The effects of temporary workers on firms' financial performance: evidence from China. *Applied Economics Letters* 2021 doi: 10.1080/13504851.2021.2014393
12. Gossett LM. Falling between the Cracks: Control and Communication Challenges of a Temporary Workforce. *Management Communication Quarterly* 2006;19(3):376-415. doi: 10.1177/0893318905280327
13. Quinlan M, Bohle P. Contingent work and occupational safety. In: Barling J, Frone MR, eds. The psychology of workplace safety. Washington, D.C.: American Psychological Association 2004.
14. Caban-Martinez AJ, Santiago KM, Stillman J, et al. Physical Exposures, Work Tasks, and OSHA-10 Training Among Temporary and Payroll Construction Workers. *Journal of occupational and environmental medicine* 2018;60(4):e159-e65.
15. Rönnblad T, Grönholm E, Jonsson J, et al. Precarious employment and mental health: a systematic review and meta-analysis of longitudinal studies. *Scandinavian Journal of Work, Environment & Health* 2019
16. Giudici F, Morselli D. 20 Years in the world of work: A study of (nonstandard) occupational trajectories and health. *Social Science and Medicine* 2019;224:138-48. doi: 10.1016/j.socscimed.2019.02.002
17. Koranyi I, Jonsson J, Rönnblad T, et al. Precarious employment and occupational accidents and injuries–a systematic review. *Scandinavian journal of work, environment & health* 2018
18. Hünefeld L, Gerstenberg S, Hüffmeier J. Job satisfaction and mental health of temporary agency workers in Europe: a systematic review and research agenda. *Work & Stress* 2020;34(1):82-110. doi: 10.1080/02678373.2019.1567619
19. Roquelaure Y, LeManach AP, Ha C, et al. Working in temporary employment and exposure to musculoskeletal constraints. *Occupational medicine* 2012;62(7):514-18.
20. Bernhard-Oettel C, Sverke M, De Witte H. Comparing three alternative types of employment with permanent full-time work: How do employment contract and perceived job conditions relate to health complaints? *Work & Stress* 2005;19(4):301-18.

For peer review only

21. Wagenaar AF, Kompier MA, Houtman IL, et al. Employment contracts and health selection: unhealthy employees out and healthy employees in? *Journal of Occupational and Environmental Medicine* 2012;54(10):1192-200.
22. Lemanski S. Stress und gesundheit in der zeitarbeit. In: Bouncken RB, Bornewasser M, eds. Zeitarbeit und Flexibilisierung: Ergebnisse des Forschungsprojektes Flex4Work München: Hampp. 2012:75-107.
23. Bernhard-Oettel C, Leineweber C, Westerlund H. Staying in or switching between permanent, temporary and self-employment during 2008–2010: Associations with changing job characteristics and emotional exhaustion. 2019;40(2):215-37. doi: 10.1177/0143831x18804648
24. Imhof S, Andresen M. Unhappy with well-being research in the temporary work context: mapping review and research agenda. *The International Journal of Human Resource Management* 2018;29(1):127-64.
25. Gupta N, Heiden M, Mathiassen S, et al. Is self-reported time spent sedentary and in physical activity differentially biased by age, gender, body mass index, and low-back pain? *Scandinavian Journal of Work, Environment & Health* 2018(2):163-70. doi: 10.5271/sjweh.3693
26. Eurofound. European Quality of Life Survey 2016: Quality of life, quality of public services, and quality of society. In: Union POotE, ed. Luxembourg, 2017.
27. OECD BF. Be Flexible! Background brief on how workplace flexibility can help European employees to balance work and family: OECD Publishing, 2016.
28. EC. Telework in the EU before and after the COVID-19: where we were, where we head to. In: Comission E, ed.: European Comission, 2020.
29. Matthew D, Harley F, A. LM, et al. Ability to work from home: evidence from two surveys and implications for the labor market in the COVID-19 pandemic. *Monthly Labor Review* 2020;U.S. Bureau of labor statistics
30. Eurofound. Living, working and COVID-19,. In: Eurofound, ed. Luxembourg: Publications Office of the European Union, 2020.
31. Allen TD, Golden TD, Shockley KM. How effective is telecommuting? Assessing the status of our scientific findings. *Psychological Science in the Public Interest* 2015;16(2):40-68.
32. Cairns TD. What Will Tip the Scales for Flexible Work Arrangements—Motivation or Collaboration? *Employment Relations Today* 2013;40(2):29-33.
33. Messenger JC. Working anytime, anywhere: The evolution of Telework and its effects on the world of work. *IusLabor* 2017(3)
34. Tavits G. Tasks and Responsibilities of an Employer in a Digital Age: How to Comply with Applicable Requirements for Work Conditions. *Juridica Int'l* 2018;27:79.
35. van der Lippe T, Lippényi Z. Co-workers working from home and individual and team performance. *New Technology, Work and Employment* 2020;35(1):60-79.
36. Nijp HH, Beckers DGJ, Geurts SAE, et al. Systematic review on the association between employee worktime control and work-non-work balance, health and well-being, and job-related outcomes. *Scandinavian Journal of Work, Environment & Health* 2012;38(4):299-313.
37. Currie J, Eveline J. E-technology and work/life balance for academics with young children. *Higher Education* 2011;62(4):533-50.
38. Golden TD. The role of relationships in understanding telecommuter satisfaction. *Journal of Organizational Behavior* 2006;27(3):319-40.
39. Mellner C. After-hours availability expectations, work-related smartphone use during leisure, and psychological detachment. *International Journal of Workplace Health Management* 2016;9(2):146-64. doi: 10.1108/IJWHM-07-2015-0050
40. Nijp HH, Beckers DGJ, van de Voorde K, et al. Effects of new ways of working on work hours and work location, health and job-related outcomes. *Chronobiology International* 2016;33(6):604-18. doi: 10.3109/07420528.2016.1167731

For peer review only - http://bmjopen.bmj.com/site/about/guidelines.xhtml

41. Čiarnienė R, Vienažindienė M, Adamonienė R. Implementation of Flexible Work Arrangements for Sustainable Development. *European Journal of Sustainable Development* 2018;7(4):11. doi: 10.14207/ejsd.2018.v7n4p11
42. Choudhury P, Foroughi C, Larson B. Work-from-anywhere: The productivity effects of geographic flexibility. 2021;42(4):655-83. doi: https://doi.org/10.1002/smj.3251
43. Emerson E, Hatton C, Robertson J, et al. The association between non-standard employment, job insecurity and health among British adults with and without intellectual impairments: Cohort study. *SSM - Population Health* 2018;4:197-205. doi: https://doi.org/10.1016/j.ssmph.2018.02.003
44. Monteiro NP, Straume OR, Valente M. When does remote electronic access (not) boost productivity? Longitudinal evidence from Portugal. *Information Economics and Policy* 2021;56:100923. doi: https://doi.org/10.1016/j.infoecopol.2021.100923
45. Sweden S. Longitudinal integrated database for health insurance and labour market studies 2021 [Available from: https://scb.se/en/services/ordering-data-and-statistics/guidance-for-researchers-and-universities/vilka-mikrodata-finns/longitudinella-register/longitudinal-integrated-database-for-health-insurance-and-labour-market-studies-lisa/.
46. Berthelsen H, Westerlund H, Bergström G, et al. Validation of the Copenhagen Psychosocial Questionnaire Version III and establishment of benchmarks for psychosocial risk management in Sweden. *International journal of environmental research and public health* 2020;17(9):3179.
47. Kuorinka I, Jonsson B, Kilbom A, et al. Standardised Nordic questionnaires for the analysis of musculoskeletal symptoms. *Applied ergonomics* 1987;18(3):233-37.
48. Nordin M, Åkerstedt T, Nordin S. Psychometric evaluation and normative data for the Karolinska Sleep Questionnaire. *Sleep and Biological Rhythms* 2013;11(4):216-26.
49. Halford S. Hybrid workspace: re-spatialisations of work, organisation and management. 2005;20(1):19-33. doi: https://doi.org/10.1111/j.1468-005X.2005.00141.x
50. Sweden S. Labour Force Surveys 2021 [Available from: https://scb.se/en/finding-statistics/statistics-by-subject-area/labour-market/labour-force-surveys/labour-force-surveys-lfs/.
51. Aboagye E, Jensen I, Bergström G, et al. Validity and test–retest reliability of an at-work production loss instrument. *Occupational Medicine* 2016;66(5):377-82. doi: 10.1093/occmed/kqw021 %J Occupational Medicine
52. Lohela Karlsson M, Bergström G, Björklund C, et al. Measuring Production Loss due to Health and Work Environment Problems: Construct Validity and Implications. *Journal of Occupational and Environmental Medicine* 2013;55(12)
53. Aronsson G, Gustafsson K. Sickness Presenteeism: Prevalence, Attendance-Pressure Factors, and an Outline of a Model for Research. 2005;47(9):958-66. doi: 10.1097/01.jom.0000177219.75677.17
54. Sullivan M, Karlsson J. The Swedish SF-36 Health Survey III. Evaluation of Criterion-Based Validity: Results from Normative Population. *Journal of Clinical Epidemiology* 1998;51(11):1105-13. doi: 10.1016/S0895-4356(98)00102-4
55. Topp CW, Østergaard SD, Søndergaard S, et al. The WHO-5 Well-Being Index: a systematic review of the literature. *Psychotherapy and psychosomatics* 2015;84(3):167-76.
56. Stevens ML, Crowley P, Garde AH, et al. Validation of a short-form version of the Danish need for recovery scale against the full scale. *International journal of environmental research and public health* 2019;16(13):2334.
57. Ilmarinen J. The work ability index (WAI). *Occupational medicine* 2007;57(2):160-60.
58. Bjärntoft S, Hallman DM, Mathiassen SE, et al. Occupational and Individual Determinants of Work-life Balance among Office Workers with Flexible Work Arrangements. *Int J Environ Res Public Health* 2020;17(4) doi: 10.3390/ijerph17041418 [published Online First: 2020/02/27]

For peer review only - http://bmjopen.bmj.com/site/about/guidelines.xhtml

59. Carlson DS, Kacmar KM, Williams LJ. Construction and Initial Validation of a Multidimensional Measure of Work–Family Conflict. *Journal of Vocational Behavior* 2000;56(2):249-76. doi: https://doi.org/10.1006/jvbe.1999.1713
60. Arapovic-Johansson B, Wåhlin C, Kwak L, et al. Work-related stress assessed by a text message single-item stress question. 2017;67(8):601-08.
61. Wiktorin C, Vingård E, Mortimer M, et al. Interview versus questionnaire for assessing physical loads in the population-based MUSIC-Norrtälje study. *American journal of industrial medicine* 1999;35(5):441-55.
62. Hagströmer M, Oja P, Sjöström M. The International Physical Activity Questionnaire (IPAQ): a study of concurrent and construct validity. *Public Health Nutrition* 2006;9(6):755-62. doi: 10.1079/PHN2005898 [published Online First: 2007/01/02]
63. Borg E, Kaijser L. A comparison between three rating scales for perceived exertion and two different work tests. *Scandinavian journal of medicine & science in sports* 2006;16(1):57-69.
64. Berthelsen H, Muhonen T, Bergström G, et al. Benchmarks for Evidence-Based Risk Assessment with the Swedish Version of the 4-Item Psychosocial Safety Climate Scale. *International Journal of Environmental Research and Public Health* 2020;17(22):8675.
65. Stemland I, Ingebrigtsen J, Christiansen CS, et al. Validity of the Acti4 method for detection of physical activity types in free-living settings: comparison with video analysis AU - Stemland, Ingunn. *Ergonomics* 2015;58(6):953-65. doi: 10.1080/00140139.2014.998724
66. Heiden M, Zetterberg C, Mathiassen SE. Trunk and upper arm postures in paper mill work. *Applied Ergonomics* 2019;76:90-96. doi: https://doi.org/10.1016/j.apergo.2018.12.004
67. Hallman DM, Srinivasan D, Mathiassen SE. Short-and long-term reliability of heart rate variability indices during repetitive low-force work. *European journal of applied physiology* 2015;115(4):803-12.
68. Hallman DM, Gupta N, Heiden M, et al. Is prolonged sitting at work associated with the time course of neck–shoulder pain? A prospective study in Danish blue-collar workers. *BMJ open* 2016;6(11):e012689.
69. Hallman DM, Birk Jørgensen M, Holtermann A. On the health paradox of occupational and leisure-time physical activity using objective measurements: Effects on autonomic imbalance. *PLoS One* 2017;12(5):e0177042.
70. Laborde S, Mosley E, Thayer JF. Heart Rate Variability and Cardiac Vagal Tone in Psychophysiological Research – Recommendations for Experiment Planning, Data Analysis, and Data Reporting. *Frontiers in Psychology* 2017;8:213. doi: 10.3389/fpsyg.2017.00213
71. Aronsson G, Gustafsson K. Sickness presenteeism: prevalence, attendance-pressure factors, and an outline of a model for research. *Journal of occupational and environmental medicine* 2005;47(9):958-66.
72. Rothman KJ. Epidemiology : an introduction. New York, NY: Oxford University Press 2012.
73. Graneheim UH, Lundman BJNet. Qualitative content analysis in nursing research: concepts, procedures and measures to achieve trustworthiness. 2004;24(2):105-12.

For peer review only - http://bmjopen.bmj.com/site/about/guidelines.xhtml

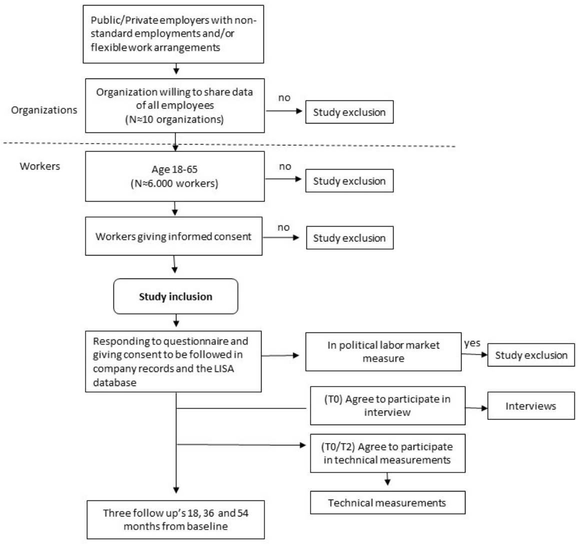

Figure 1 Recruitment of study population and data collection (intended numbers)

338x190mm (96 x 96 DPI)

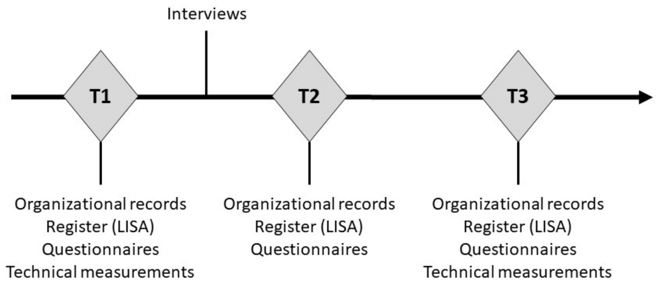

Figure 2 Flow of the data collection

338x190mm (96 x 96 DPI)

STROBE 2007 (v4) Statement—Checklist of items that should be included in reports of *cohort studies*

Section/Topic	Item #	Recommendation	Reported on page #
**Title and abstract**	1	(*a*) Indicate the study's design with a commonly used term in the title or the abstract	1
		(*b*) Provide in the abstract an informative and balanced summary of what was done and what was found	1
**Introduction**			
Background/rationale	2	Explain the scientific background and rationale for the investigation being reported	2
Objectives	3	State specific objectives, including any prespecified hypotheses	3
**Methods**			
Study design	4	Present key elements of study design early in the paper	4
Setting	5	Describe the setting, locations, and relevant dates, including periods of recruitment, exposure, follow-up, and data collection	4-5
Participants	6	(*a*) Give the eligibility criteria, and the sources and methods of selection of participants. Describe methods of follow-up	5
		(*b*) For matched studies, give matching criteria and number of exposed and unexposed	n/a
Variables	7	Clearly define all outcomes, exposures, predictors, potential confounders, and effect modifiers. Give diagnostic criteria, if applicable	6-9
Data sources/ measurement	8*	For each variable of interest, give sources of data and details of methods of assessment (measurement). Describe comparability of assessment methods if there is more than one group	5-9
Bias	9	Describe any efforts to address potential sources of bias	9-10
Study size	10	Explain how the study size was arrived at	9
Quantitative variables	11	Explain how quantitative variables were handled in the analyses. If applicable, describe which groupings were chosen and why	n/a for protocol
Statistical methods	12	(*a*) Describe all statistical methods, including those used to control for confounding	n/a for protocol
		(*b*) Describe any methods used to examine subgroups and interactions	n/a for protocol
		(*c*) Explain how missing data were addressed	n/a for protocol
		(*d*) If applicable, explain how loss to follow-up was addressed	n/a for protocol
		(*e*) Describe any sensitivity analyses	n/a for protocol
**Results**			n/a for protocol

For peer review only

Participants	13*	(a) Report numbers of individuals at each stage of study—eg numbers potentially eligible, examined for eligibility, confirmed eligible, included in the study, completing follow-up, and analysed	n/a for protocol
		(b) Give reasons for non-participation at each stage	n/a for protocol
		(c) Consider use of a flow diagram	
Descriptive data	14*	(a) Give characteristics of study participants (eg demographic, clinical, social) and information on exposures and potential confounders	n/a for protocol
		(b) Indicate number of participants with missing data for each variable of interest	n/a for protocol
		(c) Summarise follow-up time (eg, average and total amount)	n/a for protocol
Outcome data	15*	Report numbers of outcome events or summary measures over time	n/a for protocol
Main results	16	(*a*) Give unadjusted estimates and, if applicable, confounder-adjusted estimates and their precision (eg, 95% confidence interval). Make clear which confounders were adjusted for and why they were included	n/a for protocol
		(*b*) Report category boundaries when continuous variables were categorized	n/a for protocol
		(*c*) If relevant, consider translating estimates of relative risk into absolute risk for a meaningful time period	n/a for protocol
Other analyses	17	Report other analyses done—eg analyses of subgroups and interactions, and sensitivity analyses	n/a for protocol
**Discussion**			
Key results	18	Summarise key results with reference to study objectives	10
**Limitations**			10
Interpretation	20	Give a cautious overall interpretation of results considering objectives, limitations, multiplicity of analyses, results from similar studies, and other relevant evidence	n/a for protocol
Generalisability	21	Discuss the generalisability (external validity) of the study results	n/a for protocol
**Other information**			
Funding	22	Give the source of funding and the role of the funders for the present study and, if applicable, for the original study on which the present article is based	11

*Give information separately for cases and controls in case-control studies and, if applicable, for exposed and unexposed groups in cohort and cross-sectional studies.

Note: An Explanation and Elaboration article discusses each checklist item and gives methodological background and published examples of transparent reporting. The STROBE checklist is best used in conjunction with this article (freely available on the Web sites of PLoS Medicine at http://www.plosmedicine.org/, Annals of Internal Medicine at http://www.annals.org/, and Epidemiology at http://www.epidem.com/). Information on the STROBE Initiative is available at www.strobe-statement.org.

For peer review only
